# A functional peptidoglycan hydrolase characterized from T4SS in 89K pathogenicity island of epidemic *Streptococcus suis* serotype 2

**DOI:** 10.1186/1471-2180-14-73

**Published:** 2014-03-22

**Authors:** Qiu Zhong, Yan Zhao, Tian Chen, Supeng Yin, Xinyue Yao, Jing Wang, Shuguang Lu, Yinling Tan, Jiaqi Tang, Beiwen Zheng, Fuquan Hu, Ming Li

**Affiliations:** 1 Department of Microbiology, Third Military Medical University, Chongqing 400038, China; 2 Department of Scientific Research, Daping Hospital, Research Institute of Surgery, Third Military Medical University, Chongqing 400042, China; 3 PLA Research Institute of Clinical Laboratory Medicine, Nanjing general hospital of Nanjing Military command, Nanjing 210002, China; 4 State Key Laboratory for Diagnosis and Treatment of Infectious Disease, The First Affiliated Hospital, College of Medicine, Zhejiang University, Hangzhou 310003, China

**Keywords:** *Streptococcus suis* serotype 2, Streptococcal toxic shock syndrome, Type IV secretion system, Pathogenicity island, Assembly, Peptidoglycan hydrolase

## Abstract

**Background:**

*Streptococcus suis* serotype 2 (*S. suis* 2) has evolved efficient mechanisms to cause streptococcal toxic shock syndrome (STSS), which is a new emerging infectious disease linked to *S. suis*. We have previously reported that a type IV secretion system (T4SS) harbored by the specific 89K pathogenicity island (PAI) of *S. suis* 2 contributes to the development of STSS and mediates horizontal transfer of 89K. However, the 89K T4SS machinery assembly *in vivo* and *in vitro* is poorly understood, and the component acting directly to digest the bacterial cell wall needs to be identified.

**Results:**

The *virB1-89K* gene product encoded in the 89K PAI is the only one that shows similarity to the *Agrobacterium* VirB1 component and contains a conserved CHAP domain that may function in peptidoglycan hydrolysis, which makes it a plausible candidate acting as a hydrolase against the peptidoglycan cell wall to allow the assembly of the T4SS apparatus. In the current study, the CHAP domain of VirB1-89K from *S. suis* 89K PAI was cloned and over-expressed in *Escherichia coli*, and its peptidoglycan-degrading activity *in vitro* was determined. The results indicated that the VirB1-89K CHAP domain can degrade the peptidoglycan layer of bacteria. Deletion of *virB1*-*89K* reduces significantly, but does not abolish, the virulence of *S. suis* in a mouse model.

**Conclusions:**

The experimental results presented here suggested that VirB1-89K facilitates the assembly of 89K T4SS apparatus by catalyzing the degradation of the peptidoglycan cell wall, thus contributing to the pathogenesis of *S. suis* 2 infection.

## Background

*Streptococcus suis* serotype 2 (*S. suis* 2), an important zoonotic pathogen worldwide, has evolved to be a serious problem over the past two decades [1-3]. It was reported that *S. suis* 2 only causes sporadic cases of human infection with a mortality of less than 10% [4,5]. However, it emerged as the leading cause of two large-scale outbreaks of severe epidemics in China in 1998 and 2005, respectively [6]. The unusual outbreaks affected over 200 individuals and killed 52 of them [7,8]. Besides its large size and the associated high mortality rate, these two outbreaks are unique in that a large proportion of patients were victim to streptococcal toxic shock syndrome (STSS) [7]. Before that, STSS has been limited to disease caused by the group A streptococcus [9], *S. suis* (nongroup A) has not previously been linked to STSS.

To get insight into the high virulence of the *S. suis* isolates emerged in China, we previously decoded the whole genomic sequence of two epidemic strains (98HAH12 and 05ZYH33) isolated from the 1998 and 2005 Chinese outbreaks respectively, and identified a pathogenicity island (PAI) designated 89K that is specific for Chinese outbreak isolates [10,11]. Subsequently, we provided genetic evidence showing that an 89K-borne type IV secretion system (T4SS) forms an important pathway for horizontal transfer of 89K and secretion of some unknown pathogenic effectors that are responsible for STSS caused by the highly virulent *S. suis* 2 strains [12,13]. However, the 89K T4SS assembly process *in vivo* and *in vitro* remains largely unknown.

There has long been a general lack of knowledge of T4SS functions and cellular localization in gram-positive bacteria [14]. It has been suggested that the assembly processes must be similar to or even simpler than those in gram-negative bacteria [15,16]. In the well-characterized model for the *Agrobacterium tumefaciens* VirB/D T4SS, the VirB1 component functions as a lytic transglycosylase that can digest the peptidoglycan layer of cell wall, thus facilitating the assembly of envelope-spanning protein complex of T4SS under temporal and spatial control [17,18]. Among the single operon composed of 15 genes that encodes the functional T4SS in *S. suis* 89K PAI, only the *virB1*-*89K* gene product shows similarity to the *Agrobacterium* VirB1 component and contains a conserved cysteine, histidine-dependent amidohydrolases/peptidases (CHAP) domain that may function in peptidoglycan hydrolysis [19]. We once proposed that VirB1-89K should function to punch holes in the peptidoglycan cell wall to allow the assembly of the T4SS apparatus [12]. However, we did not provide direct evidence to support this hypothesis.

In the present study, therefore, we expressed and purified the CHAP domain of VirB1-89K in *Escherichia coli*, and tested its putative peptidoglycan hydrolysis activity *in vitro*. Furthermore, an isogenic knockout mutant of *virB1*-*89K* and its complementary strain were used in a mouse infection model to assess the contribution of VirB1-89K to the virulence of *S. suis* outbreak strain. The experimental results indicated that VirB1-89K facilitates the assembly of 89K T4SS apparatus by catalyzing the degradation of the peptidoglycan cell wall, thus contributing to the pathogenesis of T4SS in the *S. suis*.

## Results

### Characterization of the CHAP domain of VirB1-89K

**Figure 1:**
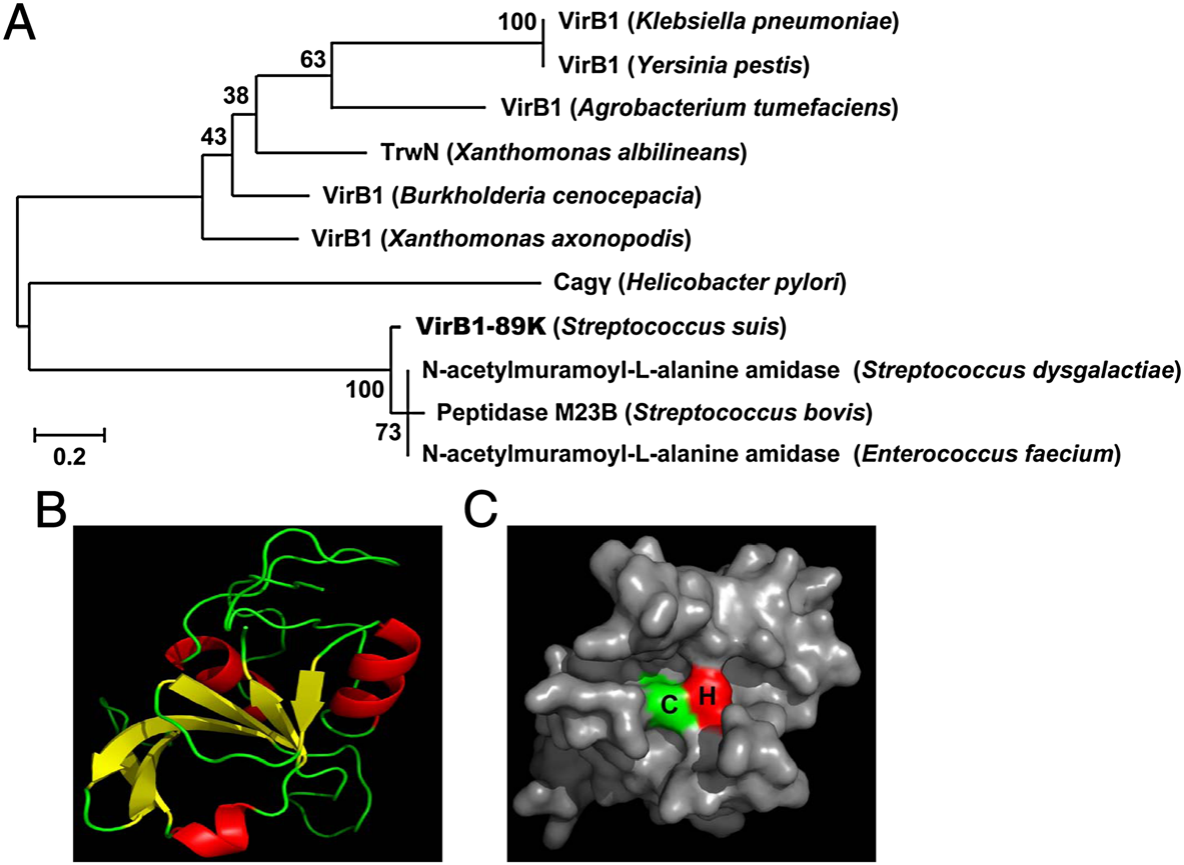
**Sequence analysis of VirB1-89K. (A)** Phylogenetic analysis of VirB1-89K. Sequence alignment and phylogenetic analysis of VirB1-89K homologs were performed using MEGA 5.1 software. Values at nodes indicate bootstrap values for 500 replicates. **(B)** Analysis of the tertiary structure of the CHAP domain of VirB1-89K by using the online server SWISS-MODEL. **(C)** Visualization of the surface active site of the CHAP domain by using PyMOLviewer, showing the cysteine residue in green and histidine in red.

On the negative strand of the 89K PAI in the genome of *S. suis* 05ZYH33 (GenBank accession number NC_009442), peptide encoded by *05SSU0968* shows 18% similarity to the VirB1 component of the *Agrobacterium* T4SS and was designated VirB1-89K. Based on sequence analysis, VirB1-89K was predicted to contain a C-terminal CHAP domain (located between the amino acids 796 and 926) and an N-terminal transmembrane domain, but lacks a signal sequence. The CHAP domain is broadly found in proteins from bacteria, phages, archaea, and eukaryotes of the Trypanosomidae family [19,20]. It has been proposed that the CHAP domain may function mainly in peptidoglycan hydrolysis [19]. The phylogenetic analysis of VirB1-89K and its homologous proteins showed that VirB1-89K and N-acetylmuramoyl-L-alanine amidase probably originate from the same ancestor (Figure 1A).

Tertiary structure prediction showed that the CHAP domain of VirB1-89K belongs to the α + β structural class, with the N-terminal half containing 3 predicted α-helices and the C-terminal half composed of 6 predicted β-strands (Figure 1B). Protein tertiary structure modeling revealed that this CHAP domain contains an putative active center composed of a conserved cysteine and a histidine (Figure 1C), these two invariant residues form the main part of the active site of CHAP domain containing proteins [19,21,22]. These results together with the above phylogeny analysis suggested that VirB1-89K may be an N-acetylmuramyl-L-alanine amidase.

### Expression and purification of the CHAP domain of VirB1-89K

**Figure 2:**
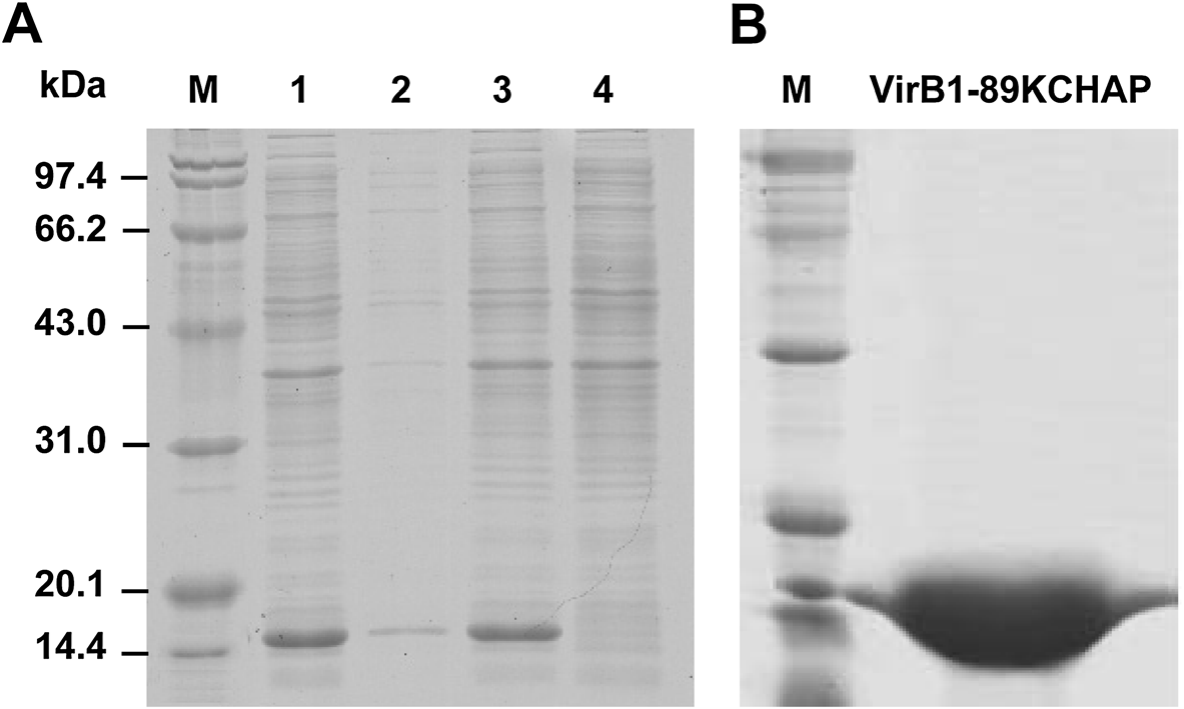
**Over-expression and purification of VirB1-89KCHAP. (A)** SDS-PAGE analysis (12%) of the interest VirB1-89KCHAP protein expressed in *E. coli*. Lanes: 1, total crude extracts after IPTG induction; 2, sonicated supernatant of induced cells; 3, sonicated sediment of induced cells; 4, noninduced cells. Protein size marker is indicated on the left. **(B)** The purified VirB1-89KCHAP protein after Ni^+^ affinity chromatography and gel filtration.

To figure out the function of VirB1-89K during the assembly of 89K T4SS apparatus, a 411 bp DNA fragment containing the CHAP domain of VirB1-89K was cloned and over-expressed in *E. coli* as a C-terminally His_6_-tagged protein. The protein of interest was designated VirB1-89KCHAP. We found VirB1-89KCHAP was efficiently expressed after induction at 16°C (Figure 2A). The molecular mass of the expressed recombinant protein agreed well with a predicted size of 15.4 kDa. Although a majority of the VirB1-89KCHAP protein was present in the inclusion body fractions of crude cell lysates, sufficient soluble material was produced to recover useful amounts of active protein. Highly purified protein (>95% homogeneity) was prepared by Ni^+^ affinity chromatography and gel filtration (Figure 2B). N-terminal sequencing results confirmed that the produced protein was indeed the CHAP domain of VirB1-89K.

### Lytic activity and biochemical characterization of VirB1-89KCHAP

**Figure 3:**
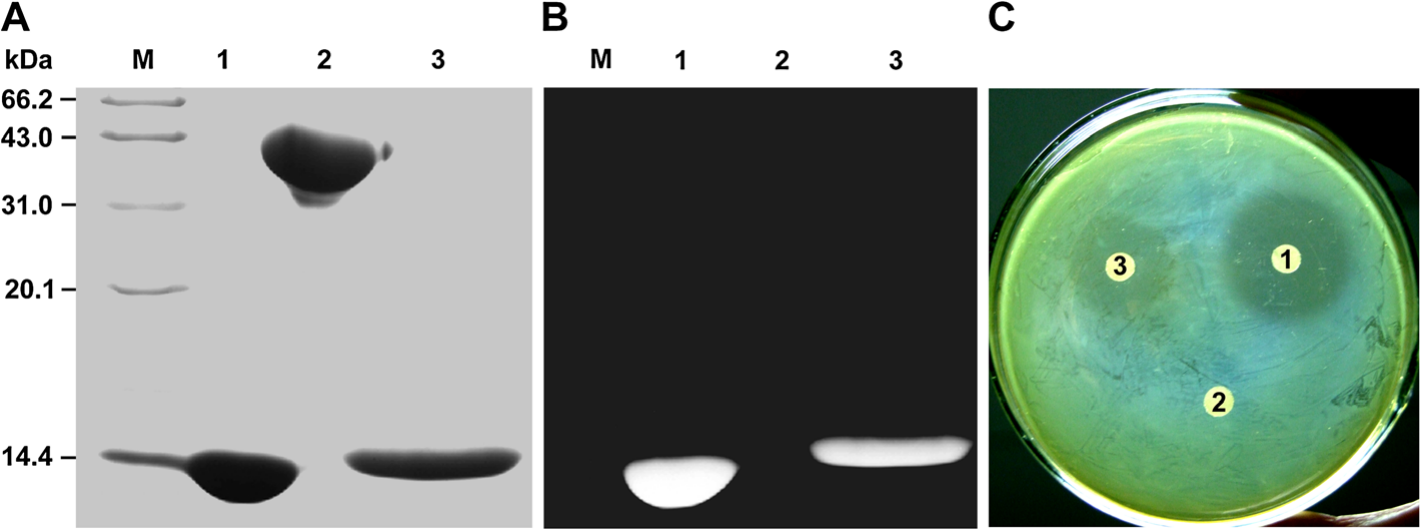
**Lytic activity detection of VirB1-89KCHAP.** Zymography analysis of peptidoglycan hydrolase activity of VirB1-89KCHAP. The gel was stained with Coomassie blue **(A)** and then overstained with Methylene blue **(B)**. **(C)** Bacteriostatic activity of VirB1-89KCHAP. Proteins used: 1, hen egg white lysozyme; 2, BSA; 3, VirB1-89KCHAP.

To determine the muramidase activity of the purified VirB1-89KCHAP protein, peptidoglycan hydrolase activity was analyzed by using zymography with *S. suis* peptidoglycan as substrate. After SDS-PAGE, the positive control hen egg white lysozyme, the negative control BSA protein, and the VirB1-89KCHAP protein could be seen after staining with Coomassie blue (Figure 3A). The gel was then stained with methylene blue to detect peptidoglycan hydrolase activity as a clear zone against a dark blue background. We noticed that VirB1-89KCHAP exhibited apparent enzyme activity as the positive control did, while the negative control BSA did not (Figure 3B). These zymography data suggested that the VirB1-89KCHAP protein could solubilize the cell wall of *S. suis* 2.

In another set of experiments, the bacteriostatic activity of VirB1-89KCHAP was determined with slip diffusion method to confirm its peptidoglycan hydrolase activity. We found that both the VirB1-89KCHAP protein and the hen egg white lysozyme could suppress the growth of *S. suis* 2, while the BSA control could not (Figure 3C).

**Figure 4:**
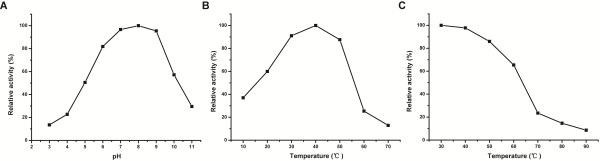
**Dynamic changes in lytic activity of VirB1-89KCHAP at different pH values or temperatures. (A)** The effect of pH on enzyme activity of VirB1-89KCHAP. **(B)** The effect of temperature on enzyme activity of VirB1-89KCHAP. **(C)** Thermostability of the VirB1-89KCHAP protein. Results shown are representative of three independent experiments.

To reveal the basic biological characteristics of VirB1-89KCHAP, we examined the optimum reaction condition of VirB1-89KCHAP by using *Micrococcus lysodeikticus* cells as substrate. Results showed that on increasing the pH, peptidoglycan hydrolase activity of VirB1-89KCHAP increases and reaches maximum at pH 8.0 (Figure 4A). When the pH exceeds 9.0, the relative activity decreased sharply. VirB1-89KCHAP functions best at an optimal temperature of 40°C. The enzyme activity rapidly declined at temperatures above 50°C and only 25% of the maximal activity was measured at 60°C (Figure 4B). From the thermal stability data, the relative activity is higher at 30°C than at 40°C, suggesting that pre-incubation of VirB1-89KCHAP at 30°C causes lower decay in relative activity compared to the enzyme pre-incubated at 40°C (Figure 4C). With increasing temperature, pre-incubation of VirB1-89KCHAP caused increasing decay in the relative activity of the enzyme.

### Role of VirB1-89K in bacterial virulence

**Figure 5:**
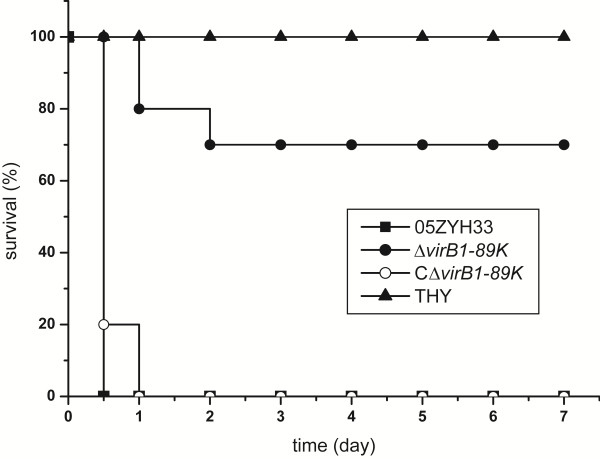
**Survival curves of mice infected with *S. suis *05ZYH33, the Δ*virB1*-*89K *mutant, the complemented strain CΔ*virB1*-*89K*, and the THY medium.** Mice (10 per group) were inoculated intraperitoneally with 10^8^ CFU bacteria. Results shown are representative of three independent experiments.

To assess the role of VirB1-89K in bacterial virulence, an isogenic knockout mutant of *virB1*-*89K* (Δ*virB1*-*89K*) constructed in our previous work and its complementary strain CΔ*virB1*-*89K* were subjected to experimental infection of mice [12]. We found that group of mice infected with the wild-type strain 05ZYH33 developed obvious clinical signs of *S. suis* infection, including rough hair coat, weight loss, depression, shivering, and suppuration of the eyes. There were no survivors at 12 hours post-infection (Figure 5). However, mice in the Δ*virB1*-*89K* mutant group were all alive at 12 hours post-infection and had a survival rate of 70% at the experimental end point of 7 days. When mice were challenged with the complemented strain, CΔ*virB1*-*89K*, data similar to those obtained with the wild-type strain were observed. In the THY control group, all mice survived without any disease symptoms during the entire experiment. These results strongly indicated that VirB1-89K is involved in the pathogenesis of Chinese epidemic *S. suis* 2 strains.

## Discussion

T4SSs are versatile devices that are found in many bacterial pathogens and secrete a wide variety of substrates, from single protein to protein-protein and protein-DNA complexes. They are generally composed of a dozen components that are organized into ATP-powered protein complexes spanning the entire cell envelope. In this macromolecular secretion apparatus, the VirB1 component can lysis cell wall peptidoglycan of the bacteria to facilitate the assembly of T4SS [23]. Many VirB1 components in gram-negative bacteria are lytic transglycosylases that can cleave the β-1,4 glycosidic bond between N-acetylglucosamine (GlcNAc) and N-acetylmuramic acid (MurNAc), with the concomitant formation of a β-1,6-anhydromuramoyl product [24-27]. In some cases, the VirB1 orthologs can be N-acetylmuramoyl-L-alanine amidases that cleave the link between N-acetylmuramoyl residues and L-amino acid residues in certain cell wall glycopeptides [28]. In this study, sequence alignment and phylogenetic analysis showed that the VirB1-89K protein may be an N-acetylmuramoyl-L-alanine amidase.

To explore the potential role of VirB1-89K in *S. suis*, we engineered the CHAP domain of VirB1-89K for heterologous expression for three reasons: i) the total length of VirB1-89K is as long as 933 amino acids located within a transmembrane region, suggesting it may be difficult to express and purify the full-length VirB1-89K protein; ii) the main function of a certain protein depends on its active domain; iii) recent studies showed that the activity of the functional domain may be higher than the full-length protein [29]. By using *S. suis* peptidoglycan as the substrate for zymogram analysis, we visually detected the muramidase activity of the purified VirB1-89KCHAP protein. In addition, the bacteriostatic activity of VirB1-89KCHAP was also observed with slip diffusion method. These data confirmed the peptidoglycan hydrolase activity of VirB1-89KCHAP, indicating the VirB1-89K component may play a crucial role in piercing the peptidoglycan layer in the cell wall of *S. suis* 2 during the assembly of the T4SS transenvelope transporter complex.

Recently, we reported that the T4SS encoded within the 89K PAI not only contributes to the development of STSS [13], but also mediates the conjugal transfer of 89K itself [12]. The transfer frequency of 89K was reduced approximately 6-fold in a *virB1*-*89K* deletion mutant (Δ*virB1*-*89K*) [12]. In this study, we found that the virulence of the Δ*virB1*-*89K* mutant was reduced to 30% compared to the wild-type level. A similar phenomenon had been reported that the *virB1* defection in *A. tumefaciens* can cause a marked reduction of virulence to 1%-10% of the wild-type level [25,30]. These results indicated that the VirB1 orthologs are important for a functional T4SS, their absence would disturb the proper assembly of the transenvelope apparatus, thus leading to unsuccessful release of the T4SS substrates.

Recent studies suggested that Cagγ, the *Helicobacter pylori* homologue of VirB1, is essential for the CagA effector translocation [31]. However, little is known about the effectors delivered by the *S. suis* T4SS that are responsible for STSS. Work currently underway in our laboratory seeks to determine these pathogenic effectors. Furthermore, our future research will focus on the difference in crystal structure between the VirB1 component in gram-negative *A. tumefaciens* and its counterpart in gram-positive *S. suis*, thus facilitating our understanding of the assembly of the T4SS apparatus in gram-positive bacteria.

## Conclusions

In summary, we characterized a functional peptidoglycan hydrolase from T4SS in the 89K PAI of Chinese epidemic *S. suis* 2. In the operon coding for the 89K T4SS, the *virB1-89K* gene product is the only one that shows similarity to the *Agrobacterium* VirB1 component and contains a conserved CHAP domain. In this work, the purified CHAP domain of VirB1-89K exhibited evident peptidoglycan-degrading and bacteriostatic activity *in vitro*. Inactivation of *virB1-89K* reduces significantly the virulence of *S. suis* in a mouse infection model. The experimental results indicated that VirB1-89K facilitates the assembly of 89K T4SS apparatus by breaking apart the peptidoglycan cell wall, thus contributing to the horizontal transfer of 89K and the pathogenesis of T4SS in *S. suis* infection.

## Methods

### Bacterial strains, plasmids, and growth conditions

The bacterial strains and plasmids used in this study are listed in Table 1. *S. suis* strains were grown in Todd-Hewitt broth (THB) supplemented with 2% yeast extract (THY). *E. coil* and *M. lysodeikticus* strains were cultured in Luria-Bertani (LB) medium at 37°C. Solid medium was prepared by the addition of 1.5% agar. When necessary, antibiotics were added at the following concentrations: spectinomycin, 100 μg/ml for both *S. suis* and *E. coli*; chloramphenicol, 5 μg/ml for *S. suis* and 10 μg/ml for *E. coli*; ampicillin, 100 μg/ml for *E. coli*.

**Table 1:** Bacterial strains and plasmids used in this study

**Strains/plasmids**	Relevant characteristics^*^	**Source/reference**
**Strains**		
***S. suis***		
05ZYH33	A highly virulent strain isolated from a dead patient with STSS	Lab collection
Δ*virB1*-*89K*	An isogenic *virB1*-*89K* mutant of strain 05ZYH33; Spc^r^	[12]
CΔ*virB1*-*89K*	Complemented strain of Δ*virB1*-*89K*; Spc^r^; Cm^r^	[12]
***M. lysodeikticus***		
ATCC4698	Suitable for substrate for the assay of lysozyme	Sigma-Aldrich
***E. coli***		
DH5α	Cloning host for maintaining the recombinant plasmids	Lab collection
BL21(DE3)	Expression host for exogenous protein production	Lab collection
**Plasmids**		
pMD19-T	Cloning vector; Amp^r^	TaKaRa
pET-21a(+)	His-tag fusion expression vector; Amp^r^	Novagen
pET21a-CHAP	A recombinant vector with the background of pET-21a(+), designed for expression of the CHAP domain of VirB1-89K; Amp^r^	This work

^*^Amp^r^, ampicillin resistant; Cm^r^, chloramphenicol resistant; Spc^r^, spectinomycin resistant.

### Bioinformatics analysis and functional prediction of VirB1-89K

Sequences were analyzed by using the DNAStar software package. Sequence alignment was performed by using BLAST at NCBI (http://www.ncbi.nlm.nih.gov/blast/). The conserved domain of VirB1-89K was analyzed using the Pfam online server (http://pfam.sanger.ac.uk/). The presence and location of signal peptide was predicted by SignalP 3.0 server (http://www.cbs.dtu.dk/services/SignalP/). The tertiary structure of the conserved domain was determined using SWISS-MODEL web server (http://swissmodel.expasy.org/) and the PyMOL viewer software. Phylogenetic analysis of VirB1-89K was conducted using the MEGA version 5.1 program.

### Cloning, expression, and purification of VirB1-89KCHAP

A 411 bp fragment encoding the CHAP domain of VirB1-89K was amplified from *S. suis* 05ZYH33 genomic DNA with the forward (5′-GAGA*CATATG*GATTTTTTTGAAAACTCTAT-3′) and the reverse (5′-GAGA*CTCGAG*TTTCGTCGTATAAGCAAAAC-3′) primers carrying the *Nde* I and *Xho* I restriction sites, respectively. The resulting PCR products were cloned into the appropriate sites of the pET-21a(+) plasmid, creating the recombinant expression vector pET21a-CHAP. A single colony of *E. coli* BL21(DE3) containing pET21a-CHAP was inoculated in LB medium and grew overnight, then diluted 1:100 into 2 L of LB medium and was grown at 37°C to an OD_600_ of 0.6. Induce cells with IPTG to a final concentration of 1 mM and grow the cultures at 16°C for an additional 10 hours. Cells were harvested by centrifugation at 6,000 rpm for 15 min, and the pellet was resuspended in 100 ml binding buffer (20 mM Na_3_PO_4_, 0.5 M NaCl, pH 8.0), and then ultrasonic treatment was performed on ice. The supernatant was collected by centrifugation, and the elution buffer (20 mM Na_3_PO_4_, 0.5 M NaCl, 0.5 M imidazole, pH 8.0) in accordance with 1:20 were added. The protein of interest (VirB1-89KCHAP) was purified on His GraviTrap column prepacked with Ni Sepharose 6 Fast Flow, then washed with binding buffer until the absorbance reaches the baseline. The target protein was eluted with elution buffer using a linear gradient. The elution was checked by SDS-PAGE (12%) and fractions containing the interest protein were further purified by gel filtration chromatography using Superdex-75 column. Peak elution fractions were analyzed by gel electrophoresis and those containing pure protein were pooled and concentrated in an Amicon apparatus (Millipore) with a 10-kDa molecular weight cutoff membrane, then stored in 0.1-ml aliquots at −80°C. The protein concentration was determined by using the Pierce BCA protein assay kit.

### Determination of the lytic activity of VirB1-89KCHAP

To determine the peptidoglycan-degrading activity of VirB1-89KCHAP, zymogram analysis was performed as described previously [32,33]. Peptidoglycan isolated and purified from *S. suis* 2 was added into 12% polyacrylamide gels to a final concentration of 100 mg/ml [24,34]. After electrophoresis, the gels were incubated at 37°C in renaturation buffer (20 mM sodium phosphate buffer, 0.1% Trition X-100, 10 mM MgCl_2_, pH 8.0) for 16 h, and then stained with 1% methylene blue containing 0.1% KOH. The deionized water was used for depolarization.

The bacteriostatic activity of VirB1-89KCHAP was determined with slip-agar diffusion method [35]. A small piece of filter paper loaded with purified VirB1-89KCHAP was placed on a 1.5% agar plate inoculated with *S. suis* 2 cells, and then bacteriostatic rings of protein-sensitive slips were generally observed after incubation and the diameters of bacteriostatic rings were measured with a vernier caliper. Hen egg white lysozyme and BSA were used as positive and negative controls, respectively.

### The effect of pH and temperature on the enzymatic activity of VirB1-89KCHAP

The effect of pH and temperature on the enzymatic activity of VirB1-89KCHAP was determined as previously described with minor modifications [31]. Purified VirB1-89KCHAP protein was added to 200 μl the dried cells of *M. lysodeikticus* as substrate. To determine the optimal pH value, the enzyme activity was monitored at 37°C with different pH values ranging from 3.0 to 11.0. The optimum temperature of the enzyme was tested at the temperature ranging from 20°C to 70°C at the optimum pH value. For the thermal stability estimation, the enzyme was pre-incubated at temperatures between 30°C and 90°C for 30 min, and the remaining activity was determined under the optimum reaction conditions.

### *In vivo* virulence studies

To determine whether the *virB1*-*89K* gene is necessary for the virulence of the highly pathogenic *S. suis* 2, experimental infection was performed as previously described [36]. Randomized groups of 10 BALB/c mice (4-week-old, female) were challenged intraperitoneally with the wild-type, the isogenic knockout mutant of *virB1*-*89K* (Δ*virB1*-*89K*), and the complementary strain CΔ*virB1*-*89K*, at a dose of 10^8^ CFU (0.1 ml of each strain) respectively. In parallel, another group of mice was injected with the same volume of THY medium as a negative control. Mice were monitored for clinical signs and survival time for 7 days. All the experiments were approved by the Laboratory Animal Welfare and Ethics Committee of the Third Mililary Medical University.

### Statistical analysis

Where appropriate, the data were analyzed using Student’s *t*-test, and a value of *P* < 0.05 was considered significant.

## Competing interests

The authors declare that they have no competing interests.

## Authors’ contributions

ML and FH conceived of the study, and JT participated in its design and coordination. QZ, YZ, TC, SY, JW, SL, and YT participated in the experiments. XY and BZ performed the sequence analysis. QZ and ML drafted the manuscript. All authors read and approved the final manuscript.
